# H3K4 Methylation Promotes Expression of Mitochondrial Dynamics Regulators to Ensure Oocyte Quality in Mice

**DOI:** 10.1002/advs.202204794

**Published:** 2023-02-23

**Authors:** Ning‐hua Mei, Shi‐meng Guo, Qi Zhou, Yi‐ran Zhang, Xiao‐zhao Liu, Ying Yin, Ximiao He, Jing Yang, Tai‐lang Yin, Li‐quan Zhou

**Affiliations:** ^1^ Institute of Reproductive Health Tongji Medical College Huazhong University of Science and Technology Wuhan Hubei 430030 China; ^2^ Reproductive Medical Center Renmin Hospital of Wuhan University & Hubei Clinic Research Center for Assisted Reproductive Technology and Embryonic Development Wuhan Hubei 430060 China; ^3^ School of Basic Medicine Tongji Medical College Huazhong University of Science and Technology Wuhan Hubei 430030 China

**Keywords:** H3K4 methylation, mitochondrial dysfunction, oocyte, oogenesis, zygotic genome activation

## Abstract

Significantly decreased H3K4 methylation in oocytes from aged mice indicates the important roles of H3K4 methylation in female reproduction. However, how H3K4 methylation regulates oocyte development remains largely unexplored. In this study, it is demonstrated that oocyte‐specific expression of dominant negative mutant H3.3‐K4M led to a decrease of the level of H3K4 methylation in mouse oocytes, resulting in reduced transcriptional activity and increased DNA methylation in oocytes, disturbed oocyte developmental potency, and fertility of female mice. The impaired expression of genes regulating mitochondrial functions in H3.3‐K4M oocytes, accompanied by mitochondrial abnormalities, is further noticed. Moreover, early embryos from H3.3‐K4M oocytes show developmental arrest and reduced zygotic genome activation. Collectively, these results show that H3K4 methylation in oocytes is critical to orchestrating gene expression profile, driving the oocyte developmental program, and ensuring oocyte quality. This study also improves understanding of how histone modifications regulate organelle dynamics in oocytes.

## Graphical abstract

To identify how H3K4 methylation is involved in oocyte development, it is demonstrated that oocyte‐specific expression of dominant negative mutant H3.3‐K4M leads to a decrease in the level of H3K4 methylation in mouse oocytes, resulting in increased DNA methylation and reduced transcriptional activities of genes like mitochondrial dynamics regulators, disturbing oocyte developmental potency, and causing female infertility.

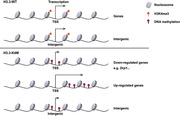

## 1 Introduction

Infertility imperils the reproductive health of women at childbearing age and is estimated to affect about 8–12% of couples worldwide,^[^ ^1^ ^]^ and the infertility rate of couples may even reach 25% in China.^[^ ^2^ ^]^ Any abnormality in the occurrence and maturation of gametes can lead to infertility.^[^ ^3^ ^]^ However, the mechanism of regulating oocyte development and maturation is still unclear. In mammals, global epigenetic reprogramming occurs during gametogenesis and early embryo development.^[^ ^4^ ^]^ Assisted reproductive technology procedures, environmental pollution, and unhealthy living habits may interfere with epigenetic reprogramming activities and impair reproductive health.^[^ ^5^ ^]^

Chromatin configuration, especially promoter‐associated epigenome, is important for gene regulation.^[^ ^6^, ^7^, ^8^ ^]^ H3K4 methylation is associated with active transcription in multiple organisms.^[^ ^9^, ^10^ ^]^ Histone lysine methyltransferases (KMTs) and histone lysine demethylases (KDMs) play important roles in regulating oogenesis and tightly regulating the methylation of H3K4 by adding and removing methyl groups.^[^ ^11^ ^]^ Mixed‐lineage leukemia 2 (MLL2) and SET domain‐containing 1 (SETD1)‐Cxxc finger protein 1 (CFP1) are the predominant H3K4 methyltransferases and ensure the developmental competence of mouse oocytes.^[^ ^12^, ^13^ ^]^ It has been reported that decreased fertility in elderly women and aged mice is closely associated with the decrease of H3K4 methylation in oocytes, revealing the critical role of H3K4 methylation in female fertility.^[^ ^4^, ^14^ ^]^

Oocyte quality is important for oocytes to mature, fertilize, and develop into viable and healthy offspring.^[^ ^15^ ^]^ Oocyte maturation includes nuclear and cytoplasmic maturation. It has been reported that the *Cxxc1*‐null oocytes exhibited reduced germinal vesicle (GV) breakdown (GVBD) and polar body 1 (PB1) emission rates during meiotic maturation, revealing the important role of H3K4 methylation on the nuclear mature of the oocyte.^[^ ^16^ ^]^ Mitochondria control many physiological functions such as energy production, metabolite synthesis, calcium signaling, cell proliferation, and death.^[^ ^17^ ^]^ Redistribution of mitochondria in the cytoplasm is an important marker of oocyte cytoplasmic maturation.^[^ ^18^ ^]^ The decrease of H3K4 methylation level by loss of CFP1 in oocytes altered the normal distribution of mitochondria, Golgi apparatus, endoplasmic reticulum, and other organelles, indicating that H3K4 methylation controls oocyte cytoplasmic maturation.^[^ ^19^ ^]^

At present, several studies have used conditional knockout mouse models to explore the functions of H3K4 methylation. However, these studies only knocked out single KMTs, and other KMTs can still exert their functions so that the inhibition of the H3K4 methylation modification level is limited. Through expression of H3.3 K‐to‐M (lysine to methionine) mutants in cells, histone methylation is suppressed at respective sites through impairing recognition ability of SET domain‐containing KMTs for KMTs activity inhibition.^[^ ^20^ ^]^

The purpose of this study is to explore the functions of H3K4 methylation in mouse oocytes by constructing H3.3‐K4M transgenic mice for inhibition of global H3K4 methylation in oocytes. We demonstrate that oocyte‐specific expression of the H3.3‐K4M mutant led to a decreased level of H3K4 methylation in mouse oocytes, impaired oogenesis, and disrupted oocyte developmental potential, resulting in female infertility.

## 2 Results

### 2.1 H3K4 Methylation Declines in Oocytes from H3.3‐K4M Transgenic Mice

**Figure 1:**
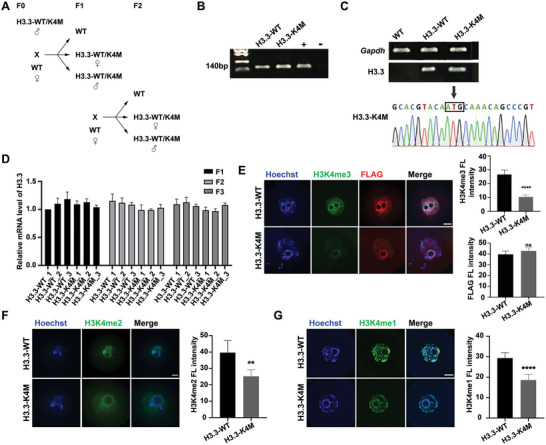
Generation and verification of transgenic mouse models. A) Schematic illustration of breeding strategy. B) Representative genotyping results of H3.3‐WT, H3.3‐K4M, and WT mice. C) RT‐PCR combined with Sanger sequencing of PCR products proved the expression of H3.3‐K4M in the ovary of H3.3‐K4M transgenic mice. D) qRT‐PCR analysis of ovaries from F1, F2, and F3 generations at P14, and H3.3‐WT and H3.3‐K4M mice showed similar levels of exogenous H3.3 transcripts in ovaries. Data are presented as Mean ± SD (*n* = 3 for each group), ^ns^ *p* > 0.05. E–G) Immunofluorescence staining and fluorescence intensity quantification of H3K4me3 (E), FLAG (E), H3K4me2 (F), and H3K4me1 (G) in GV oocytes of H3.3‐WT and H3.3‐K4M transgenic mice. Scale bar = 10 µm. Data are presented as Mean ± SD (*n* = 8 for each group), ^ns^ *p* > 0.05, ****p < 0.0001, **p < 0.01.

To study how H3K4 methylation is involved in oocyte development, we generated H3.3‐WT and H3.3‐K4M transgenic mice (Figure S1A,B, Supporting Information) and bred them by crossing with WT mice (**Figure** **1**A) and confirmed by genotyping (Figure 1B). RT‐PCR combined with Sanger sequencing proved that H3.3‐K4M was expressed in the ovary of H3.3‐K4M transgenic mice (Figure 1C). Expression of exogenous H3.3 in the ovaries of H3.3‐WT and H3.3‐K4M mice at P14 was similar, and the expression of exogenous H3.3 at F1, F2, and F3 generations of transgenic mice was also comparable, indicating that the copy number of H3.3 in H3.3‐WT and H3.3‐K4M mice was similar and could be stably inherited (Figure 1D). Western blot also showed the protein levels of H3.3‐Flag and total H3.3 were similar in H3.3‐WT and H3.3‐K4M oocytes (Figure S1C, Supporting Information). As expected, the levels of H3K4me3, H3K4me2, and H3K4me1 in GV oocytes of H3.3‐K4M transgenic mice were significantly lower than those of H3.3‐WT transgenic mice (Figure 1E–G and Figure S1C, Supporting Information), indicating that H3.3‐WT and H3.3‐K4M mice were successfully generated and were comparable. The same H3.3‐3 × Flag construct was previously used to generate transgenic mouse embryonic stem cells (mESCs),^[^ ^21^ ^]^ and ChIP‐seq of exogenous H3.3 using anti‐Flag antibody showed its enrichment at gene promoters (Figure S1D,E, Supporting Information), indicating that the H3.3‐3 × Flag construct works well for incorporation of exogenous H3.3 into the mouse genome.

### 2.2 Female H3.3‐K4M Mice were Infertile and had Less Mature Oocytes

**Figure 2:**
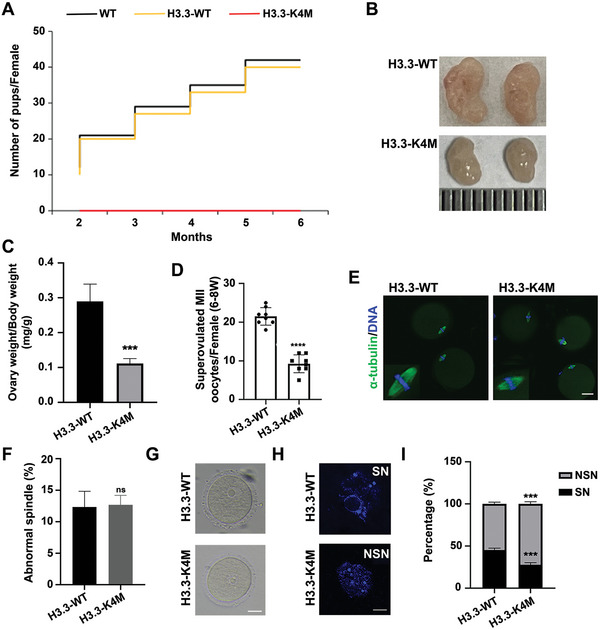
H3.3‐K4M female mice were infertile and produced less mature oocytes. A) Cumulative numbers of pups per female of indicated genotypes. Note that H3.3‐K4M female mice were infertile. B,C) Representative bright field images of ovaries (left panel) and ovary/body weight ratio (right panel) of 2‐month‐old H3.3‐WT and H3.3‐K4M mice. Data are presented as Mean ± SD (*n* = 3 for each group), ***p < 0.001. D) The numbers of MII oocytes collected per adult female mice (6‐to‐8‐week) after superovulation. Data are presented as Mean ± SD (*n* = 8 for each group), ****p < 0.0001. E) Representative images of the spindle of MII oocytes derived from H3.3‐WT and H3.3‐K4M mice. Scale bar = 10 µm. F) The percent of abnormal spindle of MII oocytes derived from H3.3‐WT and H3.3‐K4M mice. Data are presented as Mean ± SD (*n* = 100 MII oocytes from each genotype), ^ns^ *p* > 0.05. G) Representative bright field images of GV oocytes from 2‐month‐old H3.3‐WT and H3.3‐K4M mice. Scale bar = 20 µm. H) Representative images of DAPI staining displaying the chromatin configuration of NSN and SN GV oocytes. Scale bar = 10 µm. I) Quantification of percentages of NSN and SN in H3.3‐WT (*n* = 145) and H3.3‐K4M (*n* = 136) GV oocytes from adult females (6–8 weeks). Data are presented as Mean ± SD (*n* = 8 for each group), ***p < 0.001.

We found that the fertility of H3.3‐WT transgenic female mice was normal, while H3.3‐K4M transgenic female mice were infertile as no pups were obtained after crossing with stud male mice for 6 months (**Figure** **2**A). We then examined ovaries from adult H3.3‐K4M transgenic mice and they showed significantly smaller sizes than that of H3.3‐WT transgenic mice (Figure 2B,C). The numbers of GV oocytes obtained at 2 weeks, 4 weeks, 2 months, and 4 months were found to decrease gradually with increased age in H3.3‐K4M transgenic mice compared to H3.3‐WT mice (Figure S2A, Supporting Information). Moreover, collected superovulated MII oocytes were much less in adult H3.3‐K4M mice (Figure 2D), although the morphology of spindle in MII oocytes of H3.3‐K4M mice appeared normal, and there was no significant difference in the proportion of abnormal spindles between H3.3‐WT mice and H3.3‐K4M mice (Figure 2E,F). We observed no visible abnormalities in the morphology of collected GV oocytes from H3.3‐K4M mice (Figure 2G). To our surprise, the average diameter of H3.3‐K4M GV oocytes was slightly smaller than that of the H3.3‐WT GV oocytes (Figure S2B, Supporting Information). The chromatin configuration in oocytes changes during oocyte growth. There are two types of GV oocytes, the non‐surrounded nucleolus (NSN) and the surrounded nucleolus (SN). In NSN‐type oocytes, the chromatin is relaxed and the gene transcription is globally active, while in SN‐type oocytes, the chromatin is highly condensed and gathered around the nucleolus, and the gene transcription is globally silenced. NSN oocytes represent a slightly earlier developmental stage than SN oocytes and their diameters are smaller than SN oocytes. The ratio of collected GV oocytes with NSN and SN chromatin configuration showed a significant difference between H3.3‐WT and H3.3‐K4M, with about half of the oocytes being NSN type in H3.3‐WT mice and about 75% of the oocytes were NSN type in H3.3‐K4M mice (Figure 2H,I). Our further careful examination showed that the smaller average size of collected H3.3‐K4M oocytes was simply because of the increased proportion of NSN oocytes in H3.3‐K4M mice (Figure S2C, Supporting Information).

### 2.3 Oocyte Development and Maturation were Disturbed in H3.3‐K4M Transgenic Mice

**Figure 3:**
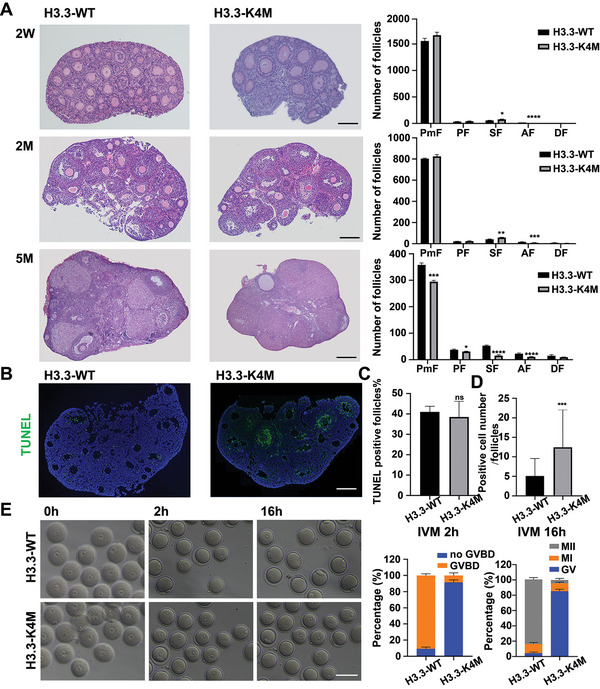
Follicle development and oocyte maturation were disturbed in H3.3‐K4M mice. A) Hematoxylin/eosin staining of the paraffin slides showing the morphologies of ovaries from 2‐week(2W), 2‐month(2M), and 5‐month(5M)‐old H3.3‐WT and H3.3‐K4M mice. Statistical analysis of the numbers of follicles in the ovaries of H3.3‐WT and H3.3‐K4M transgenic mice are shown in right. Data are presented as Mean ± SD (*n* = 3 for each group), *p < 0.05, **p < 0.01, ***p < 0.001, ****p < 0.0001. B) TUNEL assay of paraffin slides of ovaries from 2‐month‐old H3.3‐WT and H3.3‐K4M mice, Scale bar = 200 µm. C,D) Statistical analysis of the TUNEL assay was performed. Data are presented as Mean ± SD, *n* = 3 for each group, ^ns^ *p* > 0.05, ***p < 0.001. E) GVBD ratio and maturation ratio of H3.3‐WT and H3.3‐K4M oocytes after IVM for 2 or 16 h. Left panel shows representative bright field images of H3.3‐WT and H3.3‐K4M oocytes after IVM for 2 or 16 h. Scale bar = 200 µm. Right panels show the quantification of the GVBD ratio and maturation ratio for H3.3‐WT (*n* = 50 for each group) and H3.3‐K4M (*n* = 50 for each group) GV oocytes from ovaries of 6‐to‐8‐week mice.

We then ask about developmentally arrested stages of H3.3‐K4M oocytes and analyze the composition of follicles according to the classification of primordial, primary, secondary, antral, and degenerating follicles. The reduced number of total follicles in the ovaries of H3.3‐K4M mice compared to H3.3‐WT mice seems to contribute to smaller ovaries. The results of follicle counting of serial sections at different ages showed that the development of secondary follicles to antral follicles in H3.3‐K4M mice was severely disturbed (**Figure** **3**A). Notably, antral follicles were barely seen in the ovaries of 5‐month‐old H3.3‐K4M mice.

A decrease in follicle numbers is frequently associated with apoptosis, and ovarian granulosa cell apoptosis is the initiating factor leading to follicular atresia.^[^ ^22^ ^]^ TUNEL staining was therefore performed on ovarian sections to examine ovarian apoptosis. Our result showed that the apoptosis of ovarian granulosa cells in 2‐month‐old H3.3‐K4M mice was more significant than that in H3.3‐WT mice (Figure 3B–D).

Furthermore, we explored whether oocyte maturation was impaired in H3.3‐K4M mice by in vitro maturation (IVM) of GV oocytes. After 2 h of IVM, the GVBD rate of H3.3‐WT oocytes reached 90%, but most H3.3‐K4M oocytes remained in the GV stage (Figure 3E). Strikingly, after 16 h of IVM, about 80% of GV oocytes from H3.3‐WT transgenic mice developed to the MII stage, but most of the oocytes from H3.3‐K4M transgenic mice remained at the GV stage, indicating that oocyte maturation of H3.3‐K4M mice was severely disturbed (Figure 3E).

### 2.4 Global Transcriptional Activity was Inhibited in H3.3‐K4M Oocytes

**Figure 4:**
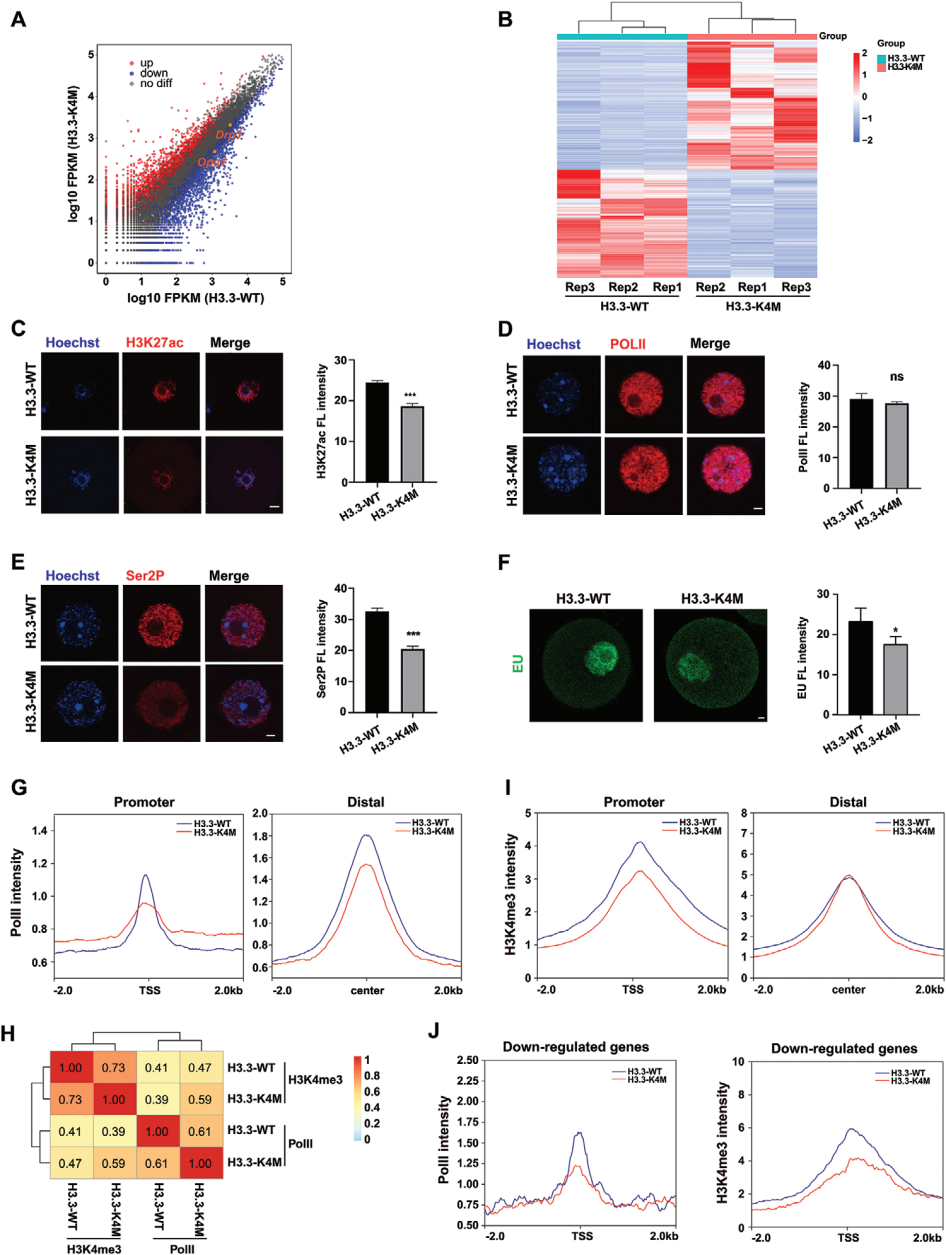
Transcriptome analysis of H3.3‐WT/K4M oocytes. A) Scatterplot displaying DEGs (downregulated, blue; upregulated, red) in H3.3‐K4M GV oocytes compared with H3.3‐WT GV oocytes, with *Drp1* and *Opa1* genes marked in orange. B) Heatmap showing differential gene expression in H3.3‐WT and H3.3‐K4M GV oocytes. C) Immunofluorescence staining and the fluorescence intensities of H3K27ac in H3.3‐WT and H3.3‐K4M GV oocytes. Scale bar = 10 µm. Data are presented as Mean ± SD, *n* = 20 GV oocytes derived from each genotype, ***p < 0.001. D) Immunofluorescence staining and the fluorescence intensities of PolII in H3.3‐WT and H3.3‐K4M GV oocytes. Scale bar = 10 µm. Data are presented as Mean ± SD, *n* = 20 GV oocytes derived from each genotype, ^ns^ *p* > 0.05. E) Immunofluorescence staining and the fluorescence intensities of Ser2P in H3.3‐WT and H3.3‐K4M GV oocytes. Scale bar = 10 µm. Data are presented as Mean ± SD, *n* = 20 GV oocytes derived from each genotype, ***p < 0.001. F) Immunofluorescence staining after EU treatment of oocytes and the fluorescence intensities of EU in H3.3‐WT and H3.3‐K4M GV oocytes. Scale bar = 20 µm. Data are presented as Mean ± SD, *n* = 10 GV oocytes derived from each genotype, *p < 0.05. G) Read density plot of PolII signals at the promoter (2 kb flanking TSSs) and distal (2 kb flanking center) regions in H3.3‐WT and H3.3‐K4M oocytes. H) Pearson correlation coefficient of H3K4me3 and PolII enrichment at gene promoters in H3.3‐WT and H3.3‐K4M oocytes. I) Read density plot of H3K4me3 signals at the promoter (2 kb flanking TSSs) and distal (2 kb flanking center) regions in H3.3‐WT and H3.3‐K4M oocytes. J) Read density plot showing PolII and H3K4me3 enrichment of down‐regulated genes at the promoter (2 kb flanking TSSs) regions in H3.3‐WT and H3.3‐K4M oocytes.

GV oocytes of 2‐month‐old H3.3‐WT and H3.3‐K4M mice were collected for transcriptome analysis to explore the disturbed genes and pathways. Generally, 2445 genes were upregulated and 2064 genes were downregulated in H3.3‐K4M oocytes (|log2Fold Change| >1 and *p* < 0.05 as a cutoff), indicating that there were significant transcriptome differences between H3.3‐WT oocytes and H3.3‐K4M oocytes (**Figure** **4**A,B and Figure S3A,B, Supporting Information). GO enrichment analysis was used to explore the functions of differentially expressed genes (DEGs). Among DEGs, mitochondrial activity and translation activity were highly enriched, and these biological processes play important roles in mouse oogenesis and maturation (Figure S3C, Supporting Information). KEGG pathway enrichment analysis of DEGs indicates that multiple metabolic pathways were significantly affected (Figure S3D, Supporting Information). Collectively, transcriptome changes indicate that the activities of mitochondria and other organelles may be impacted by RNA level in H3.3‐K4M oocytes.

It has been shown that combinations of histone codes are associated with different transcriptional activities.^[^ ^23^ ^]^ We have found that H3K4me1/2/3 levels in H3.3‐K4M oocytes were significantly lower than H3.3‐WT oocytes, and we further examined histone acetylation and transcriptional activities in H3.3‐WT and H3.3‐K4M oocytes. First, we performed immunofluorescence of H3K27ac, RNA polymerase II (PolII), and Ser2P in GV oocytes from H3.3‐WT and H3.3‐K4M mice. Ser2P, phosphorylation of the PolII C‐terminal domain on serine 2 residues, accumulates throughout the gene body and especially toward the 3′ end and is characteristic of transcriptional elongation activity.^[^ ^24^, ^25^ ^]^ We found that H3K27ac intensity was lower in H3.3‐K4M oocytes (Figure 4C), in agreement with the result that transcriptional activity was also reduced in H3.3‐K4M oocytes with no significant change of total PolII intensity (Figure 4D,E). We next verified decreased global transcription activity in H3.3‐K4M oocytes by EU staining (Figure 4F). To identify detailed alterations of transcriptional activity in oocytes, we collected growing oocytes with robust transcription activity from 2‐week‐old mice to examine the genome‐wide distribution of PolII among gene promoter and distal regions, and we found that the accumulation of PolII at the transcription start sites (TSSs) and the center of distal regions in H3.3‐K4M oocytes was significantly lower than H3.3‐WT oocytes, indicating that the overall transcriptional activity of oocytes was decreased (Figure 4G). As H3K4 methylation was altered in H3.3‐K4M oocytes and H3K4me3 is well known to be highly associated with active transcription, we then examined the genome‐wide distribution of H3K4me3. We found that the accumulation of PolII and H3K4me3 at gene promoters was indeed positively correlated (Figure 4H). We also found that the accumulation of H3K4me3 at TSSs of H3.3‐K4M oocytes was significantly lower than H3.3‐WT oocytes and narrower than that of H3.3‐WT oocytes over distal regions (Figure 4I). Further analysis showed that the accumulation of PolII and H3K4me3 over the TSS regions of the down‐regulated genes in DEGs was lower in H3.3‐K4M oocytes than in H3.3‐WT oocytes (Figure 4J). The reverse was true at genomic loci of up‐regulated genes (Figure S3E,F, Supporting Information). These results indicated that transcriptional activity alteration was correlated with H3K4me3 occupancy changes in oocytes.

### 2.5 Global DNA Methylation was Enhanced in H3.3‐K4M Oocytes

**Figure 5:**
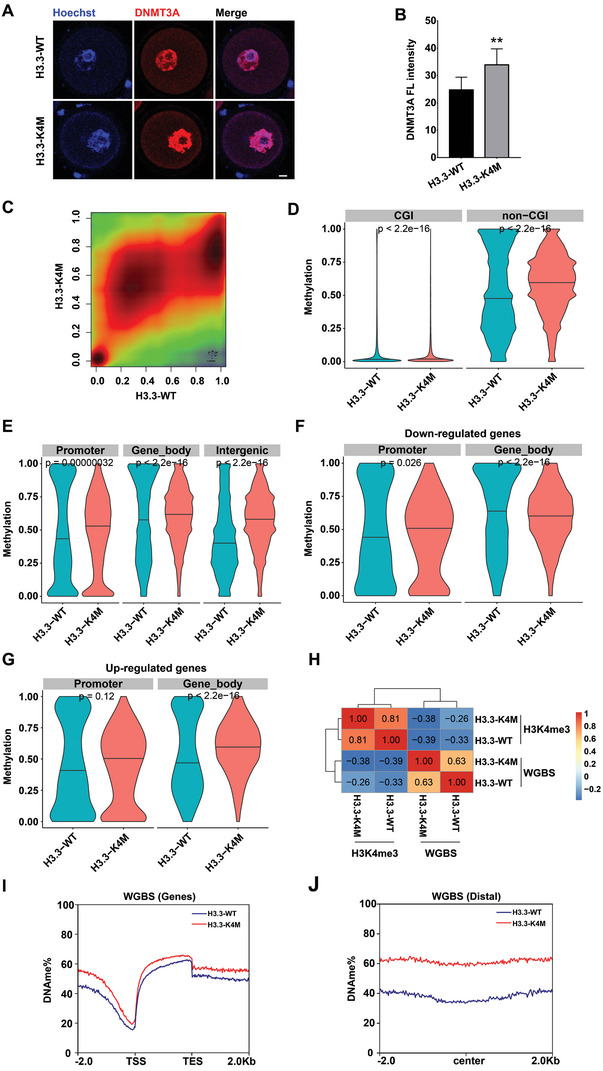
Increase of DNA methylation in H3.3‐K4M oocytes. A) Immunofluorescence staining and B) the fluorescence intensities of DNMT3A in H3.3‐WT and H3.3‐K4M GV oocytes. Scale bar = 10 µm. Data are presented as Mean ± SD, *n* = 18 GV oocytes derived from each genotype, **p < 0.01. C) Smooth scatter plot shows CpG methylation levels in H3.3‐WT and H3.3‐K4M GV oocytes. D) Violin plot shows alterations of DNA methylation at CGI and non‐CGI in H3.3‐WT and H3.3‐K4M GV oocytes. *p* values by Wilcoxon Test. E) Violin plot shows alterations of DNA methylation at gene feature regions, including promoter, gene body, and intergenic region, with black horizontal lines indicating the median. *p* values by Wilcoxon Test. F) Violin plot shows alterations of DNA methylation at gene feature regions of down‐regulated genes, including promoter, gene body, and intergenic region, with black horizontal lines indicating the median. *p* values by Wilcoxon Test. G) Violin plot shows alterations of DNA methylation at gene feature regions of up‐regulated genes, including promoter, gene body, and intergenic region, with black horizontal lines indicating the median. *p* values by Wilcoxon Test. H) Pearson correlation coefficient of H3K4me3 enrichment and DNA methylation at gene promoter regions in H3.3‐WT and H3.3‐K4M oocytes. Read density plot showing the percentage of DNA methylation at I) genic and J) distal regions in H3.3‐WT and H3.3‐K4M oocytes.

Histone modifications may have crosstalk with DNA methylation. A previous study showed that H3K4me3 could resist DNMT3A to block de novo methylation.^[^ ^26^ ^]^ In our study, we found that the protein level of DNMT3A was increased in H3.3‐K4M oocytes (**Figure** **5**A,B). To identify how DNA methylation was impacted by H3K4 methylation inhibition in oocytes, we performed low‐input whole genome bisulfite sequencing (WGBS) of control and H3.3‐K4M oocytes (Figure 5C and Figure S4A,B, Supporting Information). Analysis of WGBS data showed that there was a significant increase of DNA methylation at both CpG islands (CGIs) and non‐CGIs in H3.3‐K4M oocytes, indicating that H3K4 methylation inhibited DNA methylation activity in oocytes (Figure 5D). Our further analysis showed that DNA methylation at promoter regions was also increased upon H3K4 methylation deficiency (Figure 5E), which is in agreement with reduced PolII occupancy in H3.3‐K4M oocytes. Moreover, we noticed profound alterations of DNA methylation at the promoter (increased), gene body (increased), and intergenic (increased) regions (Figure 5E and Figure S4C, Supporting Information), suggesting crosstalk of DNA methylation status among various genomic regions. We also found that DNA methylation was reduced at gene body regions in down‐regulated genes and increased in up‐regulated genes (Figure 5F,G and Figure S4D,E, Supporting Information), which is in agreement with that DNA methylation at gene body regions facilitates gene expression.^[^ ^27^ ^]^ Correlation analysis showed that H3K4me3 enrichment and DNA methylation level were negatively correlated over the promoter and gene body regions but not intergenic regions (Figure 5H and Figure S4F,G, Supporting Information). Moreover, genome‐wide DNA methylation analysis showed reduced H3K4me3 led to increased DNA methylation at both genic and intergenic regions (Figure 5I,J). Collectively, we propose that reduced H3K4 methylation facilitated DNA methylation, leading to abnormal gene expression in H3.3‐K4M oocytes.

**Figure 6:**
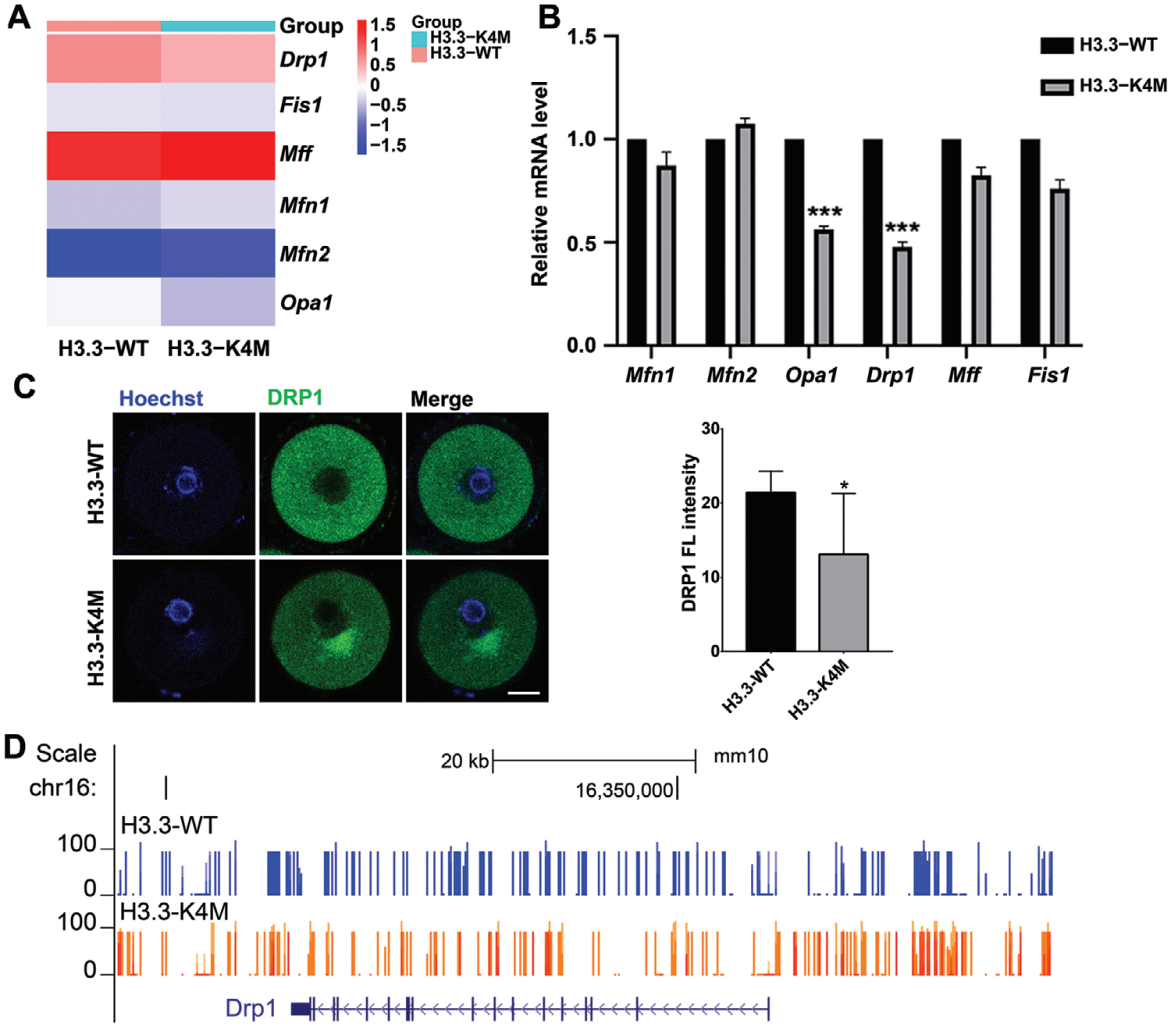
Downregulation of *Drp1* in H3.3‐K4M oocytes. A) Heatmap of mean gene expression of key mitochondrial dynamics regulators in H3.3‐WT and H3.3‐K4M GV oocytes. B) Relative expression of mitochondrial dynamics regulators validated by qRT‐PCR. C) Immunofluorescence staining and the fluorescence intensities of DRP1 in H3.3‐WT and H3.3‐K4M GV oocytes. Scale bar = 10 µm. Data are presented as Mean ± SD, *n* = 17 GV oocytes derived from each genotype, **p < 0.01. D) Visualization of DNA methylation landscape at *Drp1* locus in H3.3‐WT and H3.3‐K4M GV oocytes.

Mitochondria are important organelles that ensure oocyte development, and our RNA‐seq results showed significant enrichment of misregulated genes involved in mitochondria‐related processes. Mitochondria are highly dynamic organelles with continuous fission and fusion in the cytoplasm for regulation of their morphology, distribution, and function.^[^ ^28^ ^]^ Interestingly, our RNA‐seq result showed downregulation of Dynamic‐related protein 1 (*Drp1*) and Optic atrophy type 1 (*Opa1*) (Figure 4A), and they are two critical factors regulating mitochondrial fission and fusion and play important roles in oocyte maturation.^[^ ^29^, ^30^ ^]^ Through RNA‐seq combined with qRT‐PCR verification, we showed that expression of *Drp1* and *Opa1*, but not other mitochondrial dynamics regulators were downregulated in GV oocytes of H3.3‐K4M transgenic mice compared to H3.3‐WT transgenic mice (**Figure** **6**A,B). Further examination proved a reduction of DRP1 protein level by immunofluorescence staining and western blot in GV oocytes of H3.3‐K4M transgenic mice, while MFN1 protein level was not changed (Figure 6C and Figure S5A,B, Supporting Information). What is more, we found that the DNA methylation level was increased around the promoter region of the *Drp1* locus (Figure 6D). Therefore, a decrease of H3K4 methylation induced the enhancement of global DNA methylation, leading to the downregulation of mitochondrial dynamics regulators.

### 2.6 Mitochondrial Dysfunction in H3.3‐K4M Oocytes

**Figure 7:**
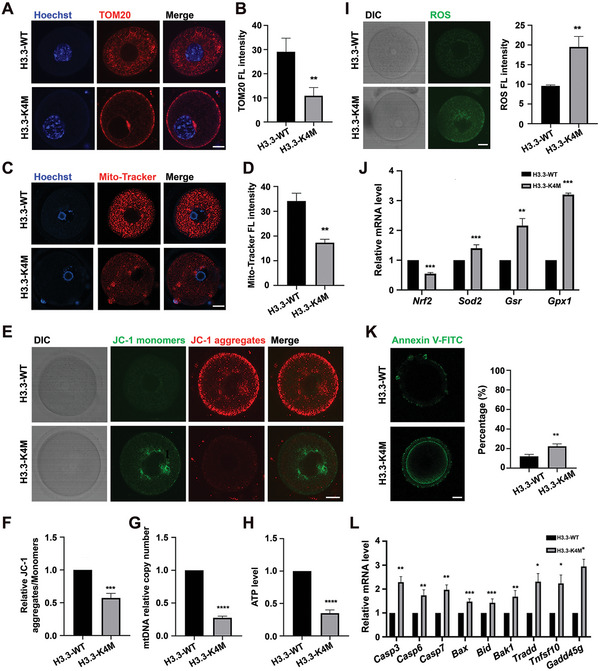
Mitochondrial dysfunction in H3.3‐K4M oocytes. A) Immunofluorescence staining of TOM20 in H3.3‐WT and H3.3‐K4M GV oocytes. Scale bar = 10 µm. B) The fluorescence intensity quantification of TOM20 in H3.3‐WT and H3.3‐K4M GV oocytes. Data are presented as Mean ± SD, *n* = 20 GV oocytes derived from each genotype, **p < 0.01. C) H3.3‐WT and H3.3‐K4M GV oocytes were labeled with Mito‐Tracker Red to visualize active mitochondria for examination of quantification and localization. Scale bar = 10 µm. D) The fluorescence intensity quantification of Mito‐Tracker Red in H3.3‐WT and H3.3‐K4M GV oocytes. Data are presented as Mean ± SD, *n* = 20 GV oocytes derived from each genotype, ** *p* <0.01. E) Representative images of mitochondrial membrane potential assessed by JC‐1 dye. Scale bar = 10 µm. F) Histogram showing the JC‐1 aggregates/monomers fluorescence ratio. Data are presented as Mean ± SD, *n* = 20 GV oocytes derived from each genotype, ***p < 0.001. G) The relative mtDNA copy numbers in H3.3‐WT and H3.3‐K4M GV oocytes. Data are presented as Mean ± SD, *n* = 20 GV oocytes derived from each genotype, ****p < 0.0001. H) Quantitative analysis of the relative ATP levels from H3.3‐WT and H3.3‐K4M GV oocytes. Data are presented as Mean ± SD, *n* = 20 GV oocytes derived from each genotype, ****p < 0.0001. I) H3.3‐WT and H3.3‐K4M GV oocytes were labeled with DCFH‐DA to visualize ROS level. Scale bar = 10 µm. Right panel shows the quantification of fluorescence intensity of ROS in H3.3‐WT and H3.3‐K4M oocytes. Data are presented as Mean ± SD, *n* = 20 GV oocytes derived from each genotype, **p < 0.01. J) Relative expression of *Nrf2*, *Sod2*, *Gsr*, and *Gpx1* in H3.3‐WT and H3.3‐K4M oocytes by qRT‐PCR. Data are presented as Mean ± SD, *n* = 3, *p < 0.05, **p < 0.01, ***p < 0.001. K) H3.3‐WT and H3.3‐K4M oocytes were labeled with Annexin‐V‐FITC to visualize apoptosis level. Scale bar = 10 µm. Right panel shows the percent of Annexin‐V‐FITC positive oocytes from H3.3‐WT and H3.3‐K4M mice. Data are presented as Mean ± SD, *n* = 20 GV oocytes derived from each genotype, **p < 0.01. L) Relative expression of pro‐apoptotic related genes in H3.3‐WT and H3.3‐K4M oocytes by qRT‐PCR. Data are presented as Mean ± SD, *n* = 3, *p < 0.05, **p < 0.01, ***p < 0.001.

We, therefore, assessed the mitochondrial functions of H3.3‐K4M oocytes. Mitochondria were labeled with TOM20 and Mito‐Tracker Red CMXRos, and the fluorescence intensity of mitochondria was globally decreased in H3.3‐K4M oocytes (**Figure** **7**A,D). Mitochondrial membrane potential was measured by JC‐1 staining, and the ratio of fluorescence intensity (aggregates/monomers) was significantly lower in H3.3‐K4M oocytes than that in H3.3‐WT oocytes (Figure 7E,F), representing mitochondrial membrane potential depolarization. We next evaluated in GV oocytes mtDNA copy number which was found to be significantly lower in H3.3‐K4M oocytes compared to H3.3‐WT oocytes (Figure 7G). Moreover, ATP content was also decreased in H3.3‐K4M oocytes (Figure 7H). These results support that the decrease of H3K4 methylation in oocytes resulted in mitochondrial dysfunction by impacting the expression of mitochondrial dynamics regulators.

Mitochondrial dysfunction was reported to be linked with reactive oxygen species (ROS) accumulation,^[^ ^31^ ^]^ and we indeed identified elevated ROS in H3.3‐K4M oocytes (Figure 7I). Through qRT‐PCR, we showed that the expression of *Nrf2* was downregulated in GV oocytes of H3.3‐K4M transgenic mice compared to H3.3‐WT transgenic mice, while *Sod2*, *Gsr*, and *Gpx1* were upregulated (Figure 7J). Numerous studies have confirmed that upregulation of *Sod2* expression causes increased H_2_O_2_ production, while downregulation of *Nrf2* expression decreases H_2_O_2_ catabolism and leads to H_2_O_2_ accumulation; increased oxidized glutathione production at higher *Gpx1* expression levels than *Gsr* can lead to higher oxidized glutathione (GSSG)/reduced glutathione (GSH) ratios and higher cellular oxidative stress levels.^[^ ^32^ ^]^ These results were in agreement with that oxidative stress level was elevated in H3.3‐K4M oocytes.

A decrease in mitochondrial membrane potential has been reported as a marker of early apoptosis, while an increase in oxidative stress level also activates the apoptotic pathway.^[^ ^33^ ^]^ Therefore, Annexin‐V‐FITC was used to examine cell apoptosis, and we found that the percentage of GV oocytes with early apoptosis was significantly higher in H3.3‐K4M oocytes than in H3.3‐WT oocytes (Figure 7K). Through qRT‐PCR, we showed that the expression of pro‐apoptotic genes was upregulated in H3.3‐K4M oocytes compared to H3.3‐WT oocytes (Figure 7L). These results confirmed that the apoptotic pathway was activated in the GV oocytes of H3.3‐K4M mice.

### 2.7 Developmental Arrest of H3.3‐K4M Early Embryos

**Figure 8:**
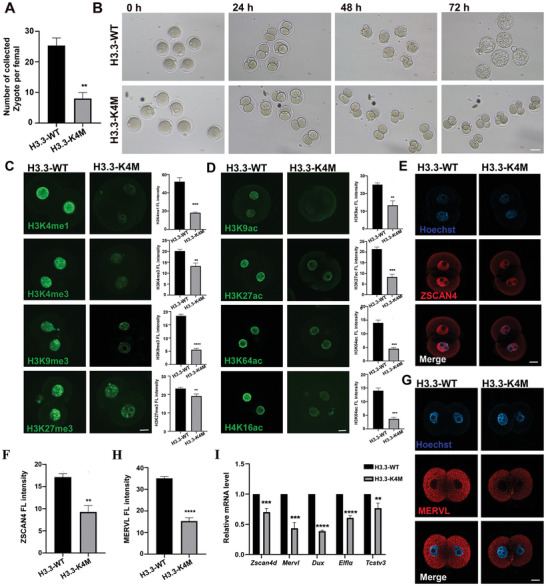
Early embryonic developmental arrest of H3.3‐K4M embryos. A) The numbers of zygotes per adult female mice (6‐to‐8‐week) after superovulation. Data are presented as Mean ± SD (*n* = 8), **p < 0.01. B) Representative bright field images of the early development of embryos by mating H3.3‐WT/K4M female with WT male for zygote collection and culturing for 24, 48, and 72, respectively. Scale bar = 50 µm. C,D) Immunofluorescence staining and fluorescence intensity quantification of histone methylation (C) and histone acetylation (D) in 2‐cell embryos derived from H3.3‐WT and H3.3‐K4M transgenic mice. Scale bar = 10 µm. Data are presented as Mean ± SD, *n* = 10 2‐cell embryos were derived from each genotype, *p < 0.05, **p < 0.01, ***p < 0.001, ****p < 0.0001. E) Immunofluorescence staining of ZSCAN4 in 2‐cell embryos from H3.3‐WT and H3.3‐K4M female. Scale bar = 10 µm. F) Quantification of fluorescence intensity of ZSCAN4 in 2‐cell embryos from H3.3‐WT and H3.3‐K4M females. Data are presented as Mean ± SD, *n* = 10 2‐cell embryos were derived from each genotype, **p < 0.01. G) Immunofluorescence staining of MERVL in 2‐cell embryos of H3.3‐WT and H3.3‐K4M female. Scale bar = 10 µm. H) Quantification of fluorescence intensity of MERVL in 2‐cell embryos of H3.3‐WT and H3.3‐K4M female. Data are presented as Mean ± SD, *n* = 10 2‐cell embryos were derived from each genotype, ****p < 0.0001. I) The relative mRNA levels of *Zscan4d*, *Mervl*, *Dux*, *Eif1a*, and *Tcstv3* by RT‐qPCR in 2‐cell embryos derived from H3.3‐WT and H3.3‐K4M female. Data are presented as Mean ± SD, *n* = 10 2‐cell embryos were derived from each genotype, **p < 0.01, ****p < 0.0001.

Although our results showed that oocyte maturation was disturbed in H3.3‐K4M mice, a small number of MII oocytes can be obtained through exogenous hormonal stimulation, and we ask whether the early development of embryos derived from H3.3‐K4M oocytes was impacted. Therefore, we mated H3.3‐WT/K4M female mice with WT male mice. As expected, we found that the number of zygotes collected from the H3.3‐K4M female was significantly less than that of the H3.3‐WT female (**Figure** **8**A). Furthermore, we found that most embryos were blocked at the 2‐to‐4‐cell stage (Figure 8B).

It has been proven that abnormal histone modifications can lead to the development deficiency of early embryos. Since H3.3‐K4M embryos were mainly arrested at the 2‐4‐cell stage, we collected H3.3‐K4M early 2‐cell embryos for immunofluorescence staining to explore the changes of histone modifications. Surprisingly, compared with H3.3‐WT early 2‐cell embryos, the levels of H3K4me1, H3K4me3, H3K9me3, H3K27me3, H3K9ac, H3K27ac, H3K64ac, and H4K16ac in H3.3‐K4M early 2‐cell embryos were all significantly decreased, while the level of total H4 was not impacted (Figure 8C,D and Figure S5C, Supporting Information). This result indicates that efficient H3K4 methylation in oocytes ensures the establishment of other histone modifications.

ZGA inhibition also impaired developmental potency. In order to explore whether the decrease of H3K4 methylation in oocytes affects ZGA event, we detected the expression of typical ZGA markers ZSCAN4 and MERVL in H3.3‐K4M early 2‐cell embryos. Our results indicated that protein levels of ZSCAN4 and MERVL were largely decreased in H3.3‐K4M early 2‐cell embryos (Figure 8E–H). In addition, qRT‐PCR was performed to validate the reduced level of ZGA transcripts, including *Zscan4d*, *Mervl*, *Dux*, *Eif1a*, and *Tcstv3*, in H3.3‐K4M early 2‐cell embryos (Figure 8I).

### 2.8 Mitochondrial Dysfunction in H3.3‐K4M Early Embryos

**Figure 9:**
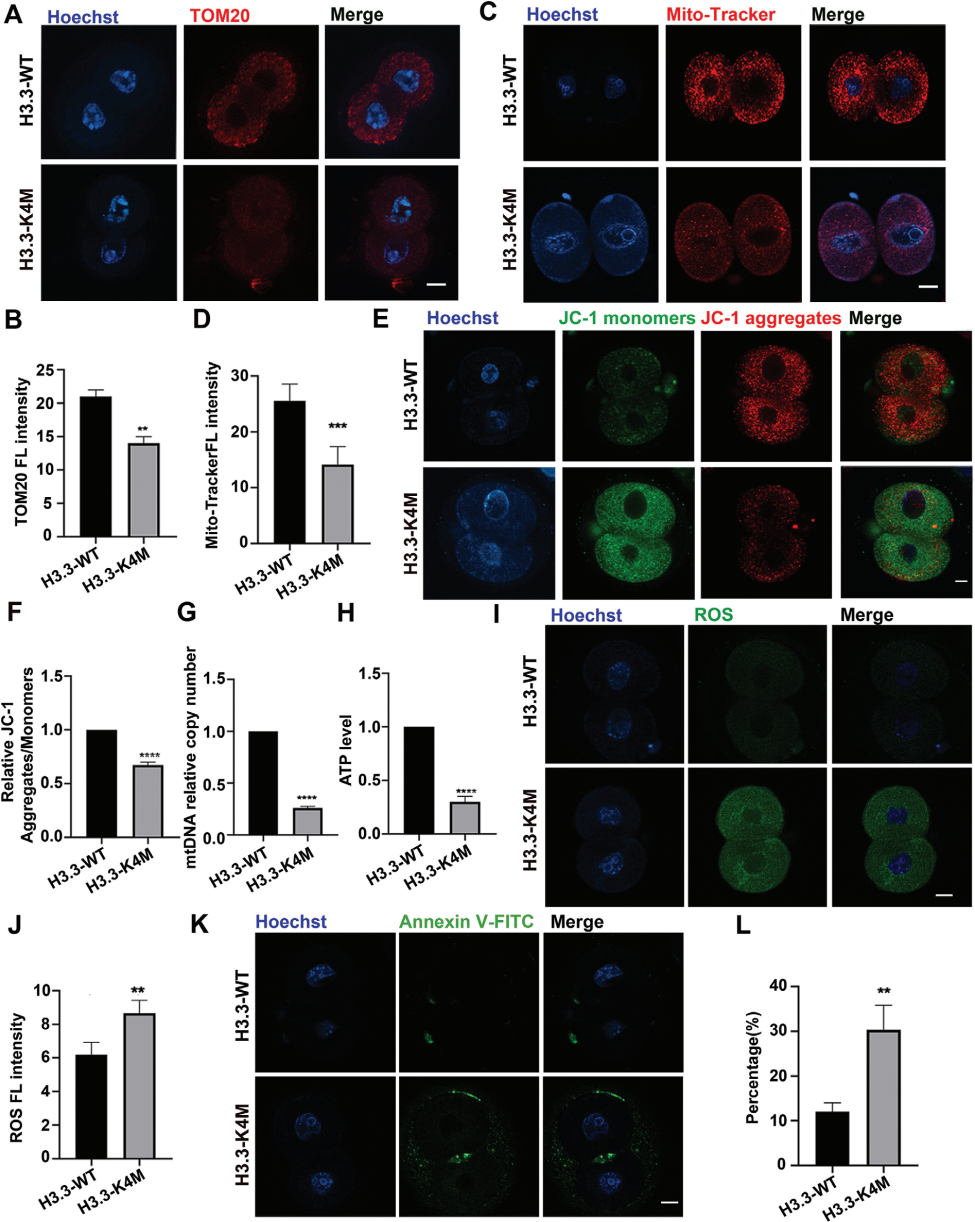
Mitochondrial dysfunction in H3.3‐K4M embryos. A) Immunofluorescence staining of TOM20 in H3.3‐WT and H3.3‐K4M 2‐cell embryos. Scale bar = 10 µm. B) Fluorescence intensity quantification of TOM20 in H3.3‐WT and H3.3‐K4M 2‐cell embryos. Data are presented as Mean ± SD, *n* = 10 2‐cell embryos were derived from each genotype, **p < 0.01. C) H3.3‐WT and H3.3‐K4M 2‐cell embryos were labeled with Mito‐Tracker Red to visualize active mitochondria. Scale bar = 10 µm. D) Fluorescence intensity quantification of Mito‐Tracker Red in H3.3‐WT and H3.3‐K4M 2‐cell embryos. Data are presented as Mean ± SD, *n* = 10 2‐cell embryos were derived from each genotype, ***p < 0.001. E) Representative images of mitochondrial membrane potential assessed by JC‐1 dye. Scale bar = 10 µm. F) Histogram showing the JC‐1 aggregates/monomers fluorescence ratio. Data are presented as Mean ± SD, *n* = 10 2‐cell embryos were derived from each genotype, ****p < 0.0001. G) The relative mtDNA copy numbers in H3.3‐WT and H3.3‐K4M 2‐cell embryos. Data are presented as Mean ± SD, *n* = 10 2‐cell embryos were derived from each genotype, ****p < 0.0001. H) Quantitative analysis of the relative ATP levels in H3.3‐WT and H3.3‐K4M 2‐cell embryos. Data are presented as Mean ± SD, *n* = 10 2‐cell embryos were derived from each genotype, ****p < 0.0001. I) H3.3‐WT and H3.3‐K4M 2‐cell embryos were labeled with DCFH‐DA to visualize ROS level. Scale bar = 10 µm. J) The fluorescence intensity of ROS in H3.3‐WT and H3.3‐K4M 2‐cell embryos. Data are presented as Mean ± SD, *n* = 10 2‐cell embryos were derived from each genotype, **p < 0.01. K) H3.3‐WT and H3.3‐K4M 2‐cell embryos were labeled with Annexin‐V‐FITC to visualize apoptosis level. Scale bar = 10 µm. L) The percent of Annexin‐V‐FITC positive 2‐cell embryos from H3.3‐WT and H3.3‐K4M female. Data are presented as Mean ± SD, *n* = 10 2‐cell embryos were derived from each genotype, **p < 0.01.

The mitochondrion is one of the key organelles driving early embryo development. First, we labeled mitochondria with Anti‐TOM20 and Mito‐Tracker Red CMXRos, and the fluorescence intensity of mitochondria was globally decreased in the H3.3‐K4M 2‐cell embryos (**Figure** **9**A–D). Mitochondrial membrane potential was also measured by JC‐1 staining. The ratio of fluorescence intensity (aggregates/monomers) was significantly lower in H3.3‐K4M 2‐cell embryos than that in H3.3‐WT (Figure 9E,F), indicating mitochondrial membrane potential depolarization. We next evaluated the number of mitochondria in 2‐cell embryos and copy number of mtDNA was found to be significantly lower in H3.3‐K4M 2‐cell embryos (Figure 9G). Moreover, ATP content was also decreased in H3.3‐K4M 2‐cell embryos (Figure 9H). The above results showed mitochondrial dysfunction in H3.3‐K4M 2‐cell embryos. Mitochondrial dysfunction may induce ROS accumulation. As expected, we found that the ROS level was increased globally in H3.3‐K4M 2‐cell embryos (Figure 9I,J). Annexin‐V‐FITC was then used to examine cell apoptosis, and we found that the percentage of 2‐cell embryos with early apoptosis was also higher in H3.3‐K4M embryos (Figure 9K,L). Therefore, deficiency of H3K4 methylation impaired mitochondrial functions in H3.3‐K4M embryos and induced ROS and apoptosis.

## 3 Discussion

Histone modifications play an important role in regulating cell cycle progression, DNA replication and repair, transcriptional activity, and chromosome stability.^[^ ^10^ ^]^ H3K4me3, H3K4me2, H3K9me3, H3K36me2, and H3K79me2 are significantly lower in GV oocytes from older mice compared to younger mice.^[^ ^34^, ^35^ ^]^ Numerous studies have found that the level of H3K4 methylation modification correlates with transcriptional activity. Moreover, there is a non‐canonical form of H3K4me3 (ncH3K4me3) signals as broad peaks at promoters and a large number of intergenic regions in oocytes, which may be involved in oocyte transcriptional silencing instead of activating.^[^ ^36^ ^]^ Previous studies showed that alteration of H3K4 methylation level by controlling H3K4 methyltransferase expression is likely to orchestrate transcriptional activity in oocytes to regulate oocyte development.^[^ ^13^, ^37^ ^]^ However, the study of H3K4 methylation functions by inhibiting histone methyltransferases has certain limitations due to the redundancy of the H3K4 methyltransferases. Based on this, we constructed H3.3‐WT and H3.3‐K4M transgenic mouse models to investigate the impact of H3K4 methylation on the developmental potential of mouse oocytes and related mechanisms.

We produced an F0 generation of transgenic mouse models through pronuclear injection and obtained two H3.3‐WT transgenic mouse strains and three H3.3‐K4M transgenic mouse strains. All the H3.3‐WT transgenic mice were fertile, while all the H3.3‐K4M transgenic mice were infertile. We selected two strains expressing the exogenous H3.3‐WT/H3.3‐K4M gene at similar levels for further experiments. We confirmed that oocyte‐specific expression of H3.3‐K4M mutants can lead to a decreased level of H3K4 methylation in mouse oocytes. In the present study, a decrease in H3K4 methylation level in oocytes led to developmental arrest in oocytes and reduced ovary size. As a consequence, H3.3‐K4M oocytes were mainly arrested at the NSN‐type oocyte stage. These H3.3‐K4M NSN‐type oocytes were difficult to complete meiosis and arrested inside the pre‐antral follicles. Growing oocytes generally undergo NSN‐to‐SN transition at PD18 to form fully‐grown oocytes, and NSN oocytes show reduced rates of meiotic maturation in vitro.^[^ ^38^, ^39^, ^40^ ^]^ Since the follicles in the H3.3‐K4M ovary were mostly arrested at the pre‐antral follicle stage, the small size of the ovary and the infertility phenotype can be attributed to the failure of oocytes to undergo NSN‐to‐SN transition.

Oocytes from the H3.3‐K4M transgenic mice showed low levels of H3K4 methylation and H3K27ac, which can lead to alteration of gene expression. This study demonstrated that the overall transcriptional activity of growing oocytes was decreased due to reduced H3K4 methylation level. For those down‐regulated genes, the accumulation of both PolII and H3K4me3 was reduced at promoter regions, and there was a significant increase of DNA methylation at promoter regions in H3.3‐K4M oocytes. Meanwhile, we found that the expression of mitochondria regulators was disturbed in H3.3‐K4M oocytes, which further led to reduced mitochondrial activity, mitochondrial membrane potential, and mtDNA copy number, and impaired mitochondrial functions. Here, mitochondrial dysfunction‐induced ROS production and oxidative stress can cause apoptosis of granulosa cells and oocytes, which further led to follicle depletion at an early developmental stage.

Defective H3K4 methylation in oocytes could also lead to early embryonic development arrest. Early 2‐cell embryos from the H3.3‐K4M transgenic mice showed changes in multiple histone modifications which may cause variations in gene expression, and reduce ZGA activity. Mitochondrial function was also impaired in early 2‐cell embryos from the H3.3‐K4M transgenic mice, with an increased level of oxidative stress and induced apoptosis in early 2‐cell embryos. Excessive ROS production is associated with the imbalance of oxidation and reduction, DNA damage, as well as developmental arrest.^[^ ^41^ ^]^ Insufficient ATP synthesis could result in decreased activity of ATP‐dependent physiological activities which may be responsible for ZGA defects in arrested 2‐cell embryos. These results suggested defective H3K4 methylation‐induced mitochondrial dysfunction and ZGA failure may be the major reasons for developmental abnormalities.

In summary, defective H3K4 methylation in oocytes impaired the development of oocytes and preimplantation embryos in mice. Reduced H3K4 methylation level resulted in mitochondrial dysfunction by disrupting mitochondrial dynamics regulators, including *Drp1* and *Opa1*, and led to excessive ROS production, increased level of oxidative stress, and induced apoptosis in oocytes and early 2‐cell embryos. Reduced transcriptional activity together with dysregulated histone modifications impaired ZGA activity and developmental potency. Our results demonstrate that defective H3K4 methylation has cytotoxic effects on oocytes and early embryonic development and provides new clues for mitochondria‐related reproductive diseases.

## 4 Experimental Section

### Generation of Transgenic Mouse Models and Mouse Breeding

The recombinant vectors ZP3 promoter‐H3.3‐WT‐3 × Flag and ZP3 promoter‐H3.3‐K4M‐3 × Flag which specifically express H3.3‐WT/K4M in oocytes were constructed by regular molecular biological techniques including enzyme digestion, enzyme ligation, and site‐directed mutagenesis. The Drosophila H3.3 sequence was used, which encoded the same protein as in mammals. The transgenic mouse models were constructed by pronuclear injection of linearized vectors for random insertion into the mouse genome. Three H3.3‐WT and two H3.3‐K4M transgenic mouse strains were obtained, respectively. Female mice from all H3.3‐WT strains were fertile, and female mice from both H3.3‐K4M strains were infertile. One H3.3‐WT strain and one H3.3‐K4M strain were selected for further examination because they had similar expression level of exogenous H3.3 in oocytes and were also proven to be stably transmitted through generations. The transgenic mice were mated with wild‐type mice for breeding and all mice were maintained on C57BL/6J × 129 background.

### Ethics Statement

All the animal procedures were approved by the Institutional Animal Care and Use Committee of Tongji Medical College, Huazhong University of Science and Technology. All animals were housed in the specific pathogen‐free (SPF) facility at Tongji Medical College, Huazhong University of Science and Technology. All experiments with mice were conducted ethically according to the Guide for the Care and Use of Laboratory Animal guidelines.

### Collection of Mouse Oocytes and Early Embryos

Growing oocytes were isolated from the ovaries of 14‐day‐old female mice by mechanical dissection using a 1 mL syringe needle. To collect GV stage oocytes from 6‐to‐8‐week‐old female mice, the ovaries were mechanically cut with ophthalmic scissors. Oocytes were sought under a stereoscopic microscope and washed three times with M2 medium (Sigma, USA). For IVM of the oocyte, GV oocytes naturally without cumulus cells were collected and washed three times with an M2 medium. Then they were transferred to an IVM medium (Aibei, China) at 37 °C with 5% CO_2_ and cultured for 16 h to collect MII oocytes.

Superovulation was performed in 6‐to‐8‐week‐old female mice by injection of 10 IU of pregnant horse serum gonadotropin (PMSG), followed by 10 IU of human chorionic gonadotropin (hCG) 48 h later. The ovulated female mice were mated with stud males in a 1:1 ratio and the vaginal plugs were examined 16 h later. Embryos were collected from the oviduct ampulla and placed in the M2 medium. Cumulus cells were digested by hyaluronidase (Sigma, USA), and embryos were washed several times with M2 medium and finally cultured in an equilibrated KSOM medium (Merck, USA) at 37 °C with 5% CO_2_.

### RNA Isolation and Quantitative Real‐Time PCR

Total RNA was extracted from ovaries, pooled oocytes (*n* = 50), and pooled embryos (*n* = 50) using TRIzol reagent (Invitrogen, USA) according to the instructions. Hifair First Strand cDNA synthesis kit (Yeason, China) was used for reverse transcription reaction. SYBR Green Master Mix (Yeason, China) was used for quantitative PCR (Quantagene q225 qPCR system). mRNA levels of genes were normalized to *Gapdh*. Each experiment was repeated at least three times independently. The primers used are shown in Table S1, Supporting Information.

### Immunofluorescence and Confocal Microscopy

Oocytes and embryos were fixed in 4% paraformaldehyde for 30 min, permeabilized with PBS/0.5% Triton X‐100 for 20 min, and blocked in PBS containing 1% BSA for 1 h. The primary antibody was incubated overnight in a wet box at 4 °C. On the second day, after washing three times with PBS, the oocytes or embryos were incubated with a secondary antibody for 2 h. DNA was stained with PBS containing 10 µg mL^−1^ Hoechst 33 342 (Invitrogen, USA). Finally, samples were examined under a confocal microscope (Zeiss LSM 780 META). The antibodies used are listed in Table S1, Supporting Information.

### Western Blot

The samples (100 GV oocytes) were lysed with 50 µL RIPA buffer (Sangon Biotech, China), and the lysates were separated in SDS‐polyacrylamide gel electrophoresis at 110 V for 60 min. Separated proteins were then transferred onto a nitrocellulose filter membrane at 350 mA for 60 min in ice. The membranes were blocked in PBS with 1% BSA for 1 h at room temperature and then incubated with primary antibody overnight at 4 °C. The membrane was incubated with peroxidase‐conjugated goat anti‐rabbit/mouse IgG (H+L) (Yeasen, China). After washing for another three times, SageCapture software was used for examination and quantification.

### Histological Analysis

Ovaries were collected and fixed in 4% paraformaldehyde overnight and then washed with PBS the next day. Samples were then embedded in paraffin, and 5 µm sections were cut and stained with hematoxylin and eosin (H&E). Images were captured by Olympus BX51 Microscope with MShot MSX2 camera.

### TUNEL Assay

TUNEL assays were performed using TUNEL Bright Green Apoptosis Detection Kit (Vazyme, China) according to the manufacturer's procedure. Sections were dewaxed, hydrated, and permeabilized with PBS/0.5% Triton X‐100, and washed three times using PBS. Then sections were blocked with equilibration buffer and incubated with the TUNEL reaction mixture for 1 h. DNA was stained with DAPI (Invitrogen, USA).

### Low‐Input RNA Sequencing and Data Analysis

A total of 5–10 GV oocytes in each group were collected for RNA seq library preparation. Generally, oocytes were collected in tubes with a lysis component and ribonuclease inhibitor. Then amplification was carried out by the Smart‐Seq2 method (Takara). Qualified libraries were loaded onto the Illumina Hiseq platform for PE150 sequencing. ≈27 m of paired‐end reads for each sample were generated. Raw reads were processed with Trim Galore (v0.6.4) to remove adaptor sequences and poor‐quality bases with “–q 20 –phred33 –stringency 5 –length 20 –paired.” Trimmed reads were aligned to the mouse reference genome (mm10) using STAR (v2.7.5b) with default settings. SAMtools (v1.3.1) was used to sort bam files by genomic coordination and make a bam file index. R package Deseq2 (v1.28.1) was used to obtain DEGs based on the reads count file obtained by STAR (v2.7.5b). Genes with an absolute log_2_FoldChange >1 and *p* adjusted <0.05 were considered as significant DEGs. Principal component analysis of RNA‐seq was performed using the prcomp function in R (v1.28.1). GO enrichment analysis was performed by the Metascape website. KEGG pathway enrichment analysis was performed using the R package Clusterprofiler (v4.6.0).

### 5‐Ethynyl Uridine Staining

Global transcription assays were performed using an EU nascent RNA detection kit (RIBOBIO, China) according to the manufacturer's instructions. Generally, GV oocytes were incubated with EU mix at 37 °C for 2 h, fixed with 4% PFA, permeabilized with 0.5% Triton X‐100 for 10 min, incubated with Apollo mix for 30 min, and washed with PBS three times. DNA was stained with PBS containing 10 µg mL^−1^ Hoechst 33 342 (Invitrogen, USA). Finally, samples were examined under a confocal microscope (Zeiss LSM 780 META).

### ChIP‐seq and Data Analysis

ChIP‐seq libraries were prepared using a Hyperactive In Situ ChIP Library Prep Kit for Illumina (pG‐Tn5) (Vazyme Biotech Co., Ltd, China) according to the manufacturer's protocol with antibodies against PolII (active motif, USA) and H3K4me3 (CST, USA). A total of 100–200 growing oocytes were used in each ChIP‐seq experiment. Qualified libraries were then loaded on the Illumina Hiseq platform for PE150 sequencing. ≈3–10 m of paired‐end reads for each sample were generated. Raw reads were processed with Trim Galore (v0.6.4) to remove adaptor sequences and poor quality bases with “–q 20 –phred33 –stringency 5 –length 20 –paired.” Trimmed reads were then aligned to the mouse reference genome (mm10) using Bowtie2 (version 2.4.2) with default parameters. Picard (version 2.26.6) was used to remove PCR duplicates. Peaks were called using MACS2 v2.2.7.1 with default parameters. The UCSC Genome Browser utility,^[^ ^42^ ^]^ bedGraphToBigWig, was used to transform the bedgraph files to bigwig files. Heatmaps of ChIP‐seq signal enrichment were generated by the Python package, deepTools v3.5.1.^[^ ^43^ ^]^ ChIP‐seq peak annotation was performed by ChIPseeker v1.34.1.^[^ ^44^ ^]^

### Low‐Input Whole Genome Bisulfite Sequencing of Oocytes and Data Analysis

A total of 5–10 GV oocytes from H3.3‐WT/K4M females were collected. The WGBS libraries were prepared using a Post‐bisulfite adapter tagging (PBAT) approach by Annoroad Gene Technology. Generally, bisulfite conversion was first performed on the cell lysate of oocytes using EZ DNA Methylation‐Gold Kit (Zymo, USA). Then oligo1 primers labeled with biotin were used to synthesize one strand. Oligo2 primers with adapter sequences were used to perform another strand synthesis followed by indexing. ≈30 m of paired‐end reads for each sample was generated by sequencing. Raw reads were processed with Trim Galore (v0.6.4) to remove adaptor sequences and poor quality bases with “–q 20 –phred33 –stringency 5 –length 20 –paired.” Trimmed reads were then aligned to the mouse reference genome (mm10) using Bismark (v0.22.3) with the parameters “‐p 6 –parallel 1 ‐N 0 ‐L 20 –quiet –pbat –un –ambiguous –bam.” SAMtools (v1.3.1) was used to sort bam files by genomic coordination and make a bam file index. PCR duplicates were removed using Picard (v2.23.3). The methylation ratio at each CpG site was constructed using bismark_methylation_extractor model with the parameters “‐p –comprehensive –no_overlap –bedgraph –counts –report –cytosine_report –gzip –buffer –size 30G.” The UCSC Genome Browser utility,^[^ ^42^ ^]^ bedGraphToBigWig, was used to transform the bedgraph files to bigwig files. UCSC genome browser was used for visualization. The methylation levels at CpG sites were first calculated by “methRead” function of R package methylKit (v1.14.2)^[^ ^45^ ^]^ with mincov = 3. Methylation across the genome was tiled with the “tileMethylCounts” function using the parameters “win.size = 1000, step. size = 1000”, then “unite” function was used to unite tiled regions with the “destrand = TRUE” parameter.

### Active Mitochondrial Staining

The oocytes and embryos were incubated with 500 nmol L^−1^ Mito Tracker Red CMXRos (Beyotime, China) at 37 °C in 5% CO_2_ for 30 min. After three washes with M2 medium, the DNA was stained with PBS containing 10 µg mL^−1^ Hoechst 33 342. Samples were then observed under confocal microscopy.

### Assay of Mitochondrial Membrane Potential

The oocytes and embryos were incubated with JC‐1 (Beyotime, China) at 37 °C in 5% CO_2_ for 30 min. Samples were observed under confocal microscopy, and membrane potential was measured as the ratio of red fluorescence (J‐aggregates) to green fluorescence (J‐monomers).

### Mitochondrial DNA Copy Number Measurement

A total of 10–20 oocytes or embryos were transferred to 20 µL lysis buffer (50 mm Tris‐HCl, 200 µg mL^−1^ proteinase K, 0.5%Triton X‐100) at 55 °C for 2 h. Real‐time PCR was performed to measure the mtDNA copy number. Each experiment was repeated at least three times independently. mtDNA levels were normalized to *Gapdh*.

### Reactive Oxygen Species Measurement

ROS Assay Kit (Beyotime, China) was used to detect total ROS in oocytes and embryos. The samples were incubated in an M2 medium containing 10 µm DCFH‐DA at 37 °C for 20 min and washed three times with M2 medium. Fluorescence signals were detected by confocal microscopy.

### ATP Measurement

ATP content was measured by the ATP determination kit (Molecular Probes, USA) according to the manufacturer's instructions. Oocytes or embryos were collected into 30 µL lysis buffer. Then, 10 µL samples were added to 96‐well plates and 100 µL standard reaction solution was added into each well subsequently. The light intensity was set as 1 in the control group, and the relative intensity of the treatment group was measured and compared to the control group.

### Oocytes and Embryos Apoptosis Analysis

The apoptosis rate was evaluated using Annexin V‐FITC Apoptosis Detection kit (Beyotime, China) according to the manufacturer's instructions. Oocytes and embryos were collected and washed with an M2 medium. Then, 195 µL binding buffer and 5 µL Annexin V‐FITC were added and incubated for 20 min in the dark. After three washes with an M2 medium, they were observed under confocal microscopy.

### Statistical Analysis

GraphPad Prism8 was used for calculation and *p*‐values were subjected to a two‐tailed Student's *t*‐test. *p*‐values were subjected to Wilcoxon Test for evaluation of DNA methylation levels. Every experiment was repeated at least three times. Significance was set at *p*‐value <0.05.

## Conflict of Interest

The authors declare no conflict of interest.

## Author Contributions

N.‐h.M., S.‐m.G. and Q.Z. contributed equally to this work. L.‐q.Z., T.‐l.Y., and J.Y. conceived the idea and revised the manuscript. N.‐h.M., S.‐m.G., and Q.Z. performed the experiments. Y.‐r.Z. helped with mouse breeding. X.‐z.L., Y.Y., and X.H. performed data analysis. N.‐h.M. wrote the initial manuscript. All authors contributed to the article and approved the final manuscript.

## Supporting information

Supporting Information

Supplemental Table 1

## Data Availability

The RNA‐seq, ChIP‐seq, and WGBS datasets in this study have been deposited to NCBI: PRJNA906363, PRJNA906499, PRJNA906481.
