# Supplementary material for: H3K4 Methylation Promotes Expression of Mitochondrial Dynamics Regulators to Ensure Oocyte Quality in Mice
Source: Adv Sci (Weinh). 2023 Feb 23;10(12):2204794. doi: 10.1002/advs.202204794 (PMC10131798; doi:10.1002/advs.202204794)
Supplement: Supplementary file 1 — Supporting Information [file ADVS-10-2204794-s001.pdf]

ADVANCED SCIENCE
Open Access

Supporting Information

for *Adv. Sci.*, DOI 10.1002/advs.202204794

H3K4 Methylation Promotes Expression of Mitochondrial Dynamics Regulators to Ensure Oocyte Quality in Mice

Ning-hua Mei, Shi-meng Guo, Qi Zhou, Yi-ran Zhang, Xiao-zhao Liu, Ying Yin, Ximiao He, Jing Yang, Tai-lang Yin* and Li-quan Zhou**

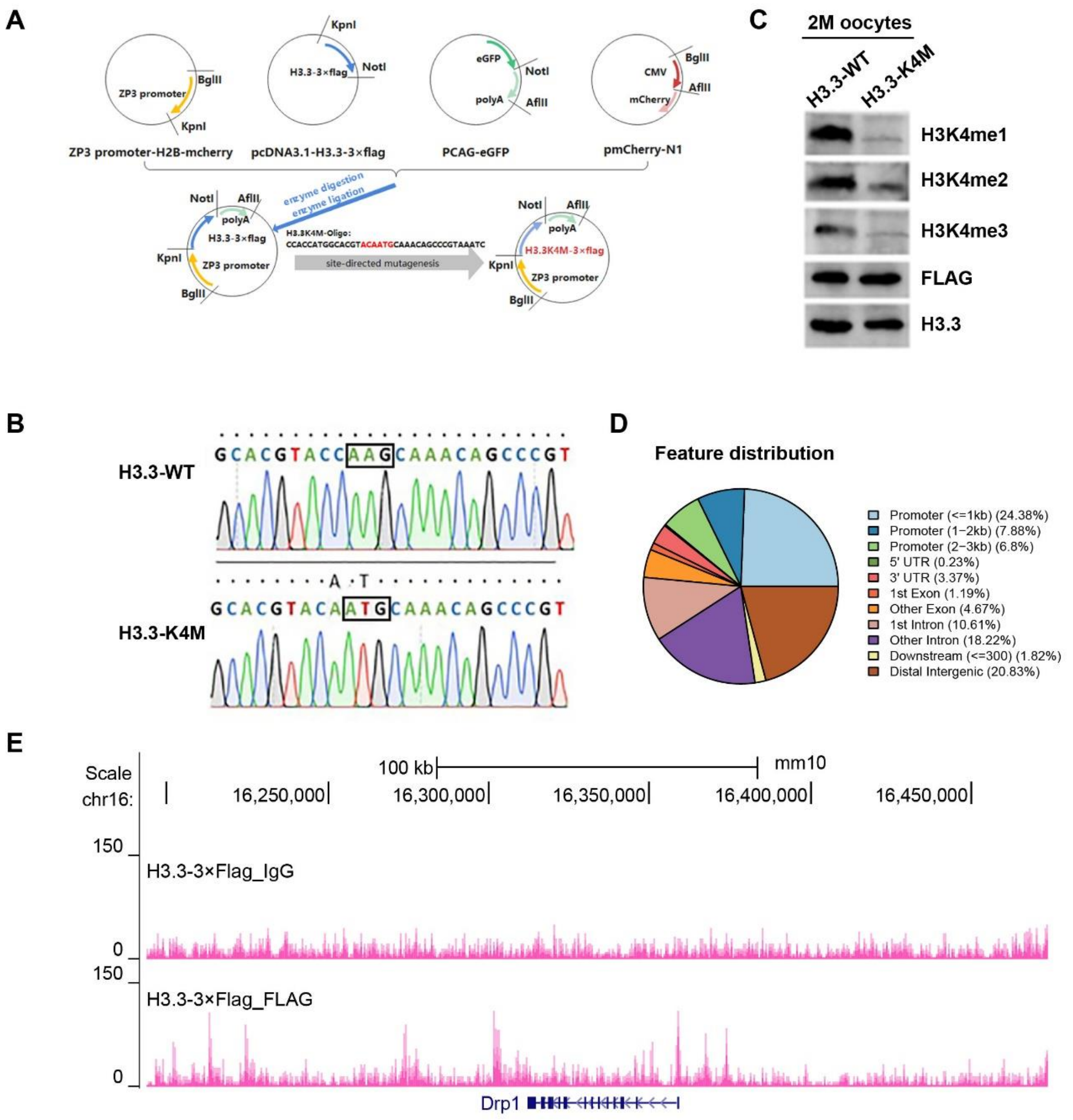

Fig. S1. Constructs of H3.3-WT/K4M for transgenic mouse production.

(A) The recombinant vectors with ZP3 promoter which drives specific expression of H3.3-WT or H3.3-K4M in oocytes were constructed by molecular biological techniques. (B) The correctness of exogenous H3.3 sequence was determined by Sanger sequencing of the constructs. (C) Western blot results showed abundance of H3K4me1, H3K4me2, H3K4me3, exogenous H3.3-Flag and total H3.3 in H3.3-WT and H3.3-K4M GV oocytes. (D) ChIP-seq of H3.3-Flag showing the feature distribution of exogenous H3.3 at mouse genome in H3.3-3×Flag transgenic mESCs (PRJNA786949). (E) Genome browser snapshots of ChIP-seq data of H3.3-3×Flag in transgenic mESC by anti-Flag antibody shows enrichment of exogenous H3.3 at *Drp1* gene promoter.

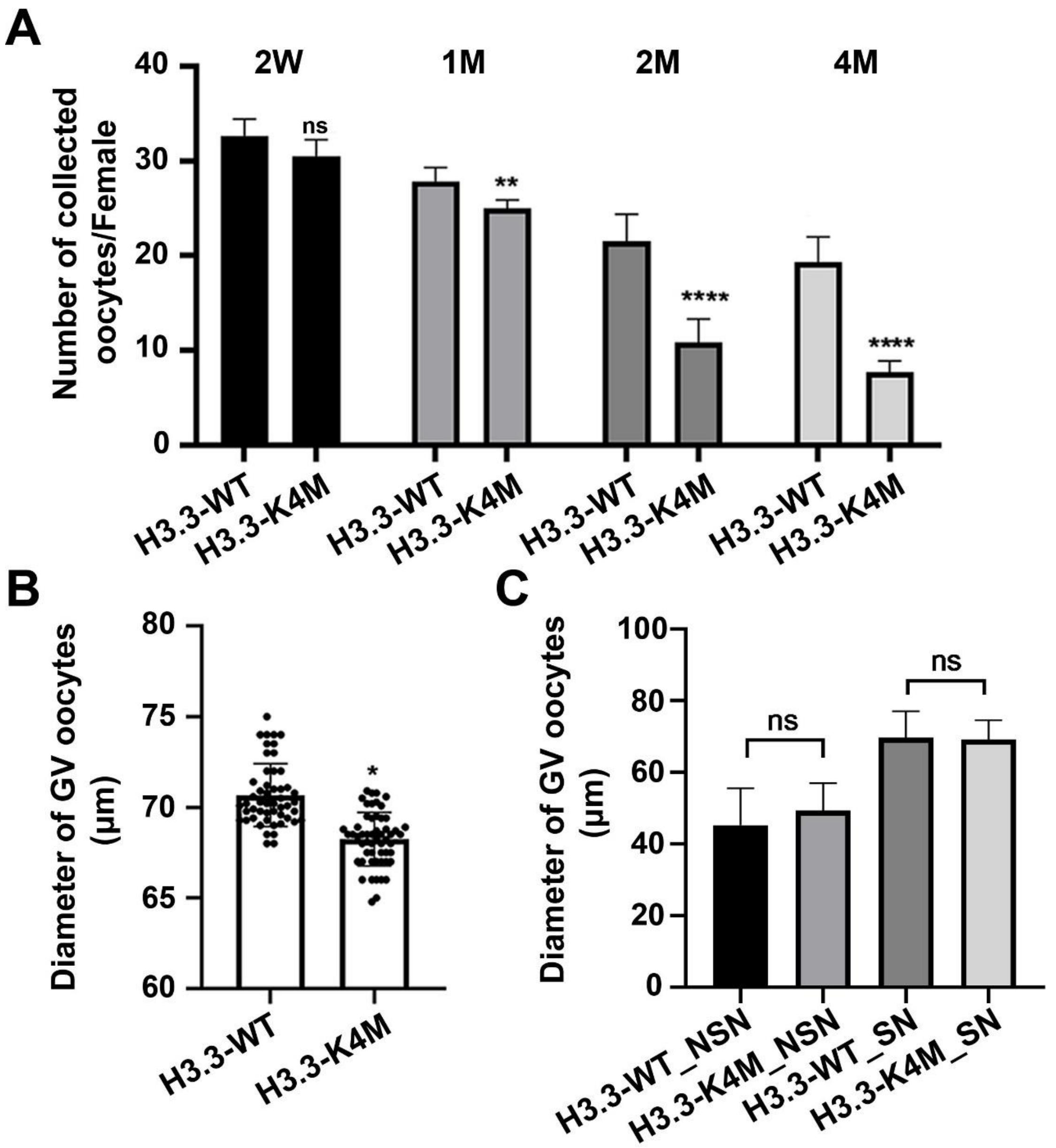

Fig. S2. Diameters of H3.3-WT and H3.3-K4M oocytes.

(A) Numbers of GV oocytes collected from each female (2-week, 1-month, 2-month and 4-month). Data are presented as Mean±SD (n=8 for each group), $^{ns}P>0.05$, ****$P<0.0001$, **$P<0.01$. (B) Quantification of oocyte size by measuring the diameters of GV oocytes (n=50 from H3.3-WT and n=50 from H3.3-K4M 6-to-8-week female mice). Data are presented as Mean±SD, *$P<0.05$. (C) Quantification of oocyte size by measuring the diameters of NSN and SN GV oocytes from H3.3-WT and H3.3-K4M transgenic mice. Data are presented as Mean±SD (n=8 for each group), $^{ns}P>0.05$.

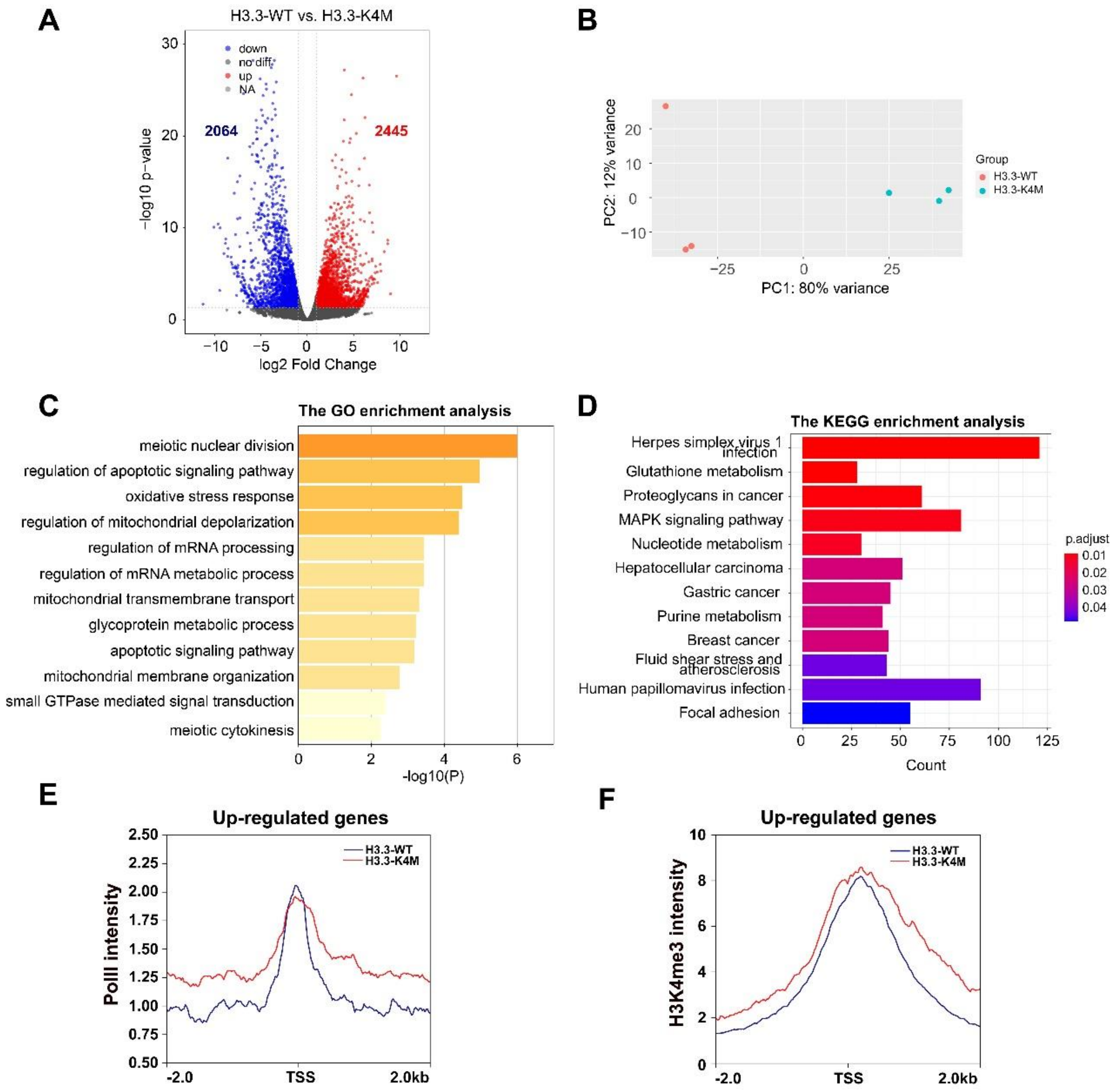

Fig. S3. Transcriptome analysis of H3.3-WT/K4M oocytes.

(A) Volcano plot displaying differentially expressed genes (downregulated, blue; upregulated, red) in H3.3-K4M GV oocytes compared with H3.3-WT. (B) PCA analysis of transcriptome of H3.3-WT and H3.3-K4M oocytes. (C) Highly enriched GO terms of differentially expressed genes in H3.3-K4M oocytes compared with H3.3-WT oocytes. (D) KEGG enrichment analysis of differentially expressed genes in H3.3-K4M oocytes compared with H3.3-WT oocytes. (E-F) Read density plot showing PolII and H3K4me3 enrichment of up-regulated genes at promoter (2 kb flanking TSSs) regions.

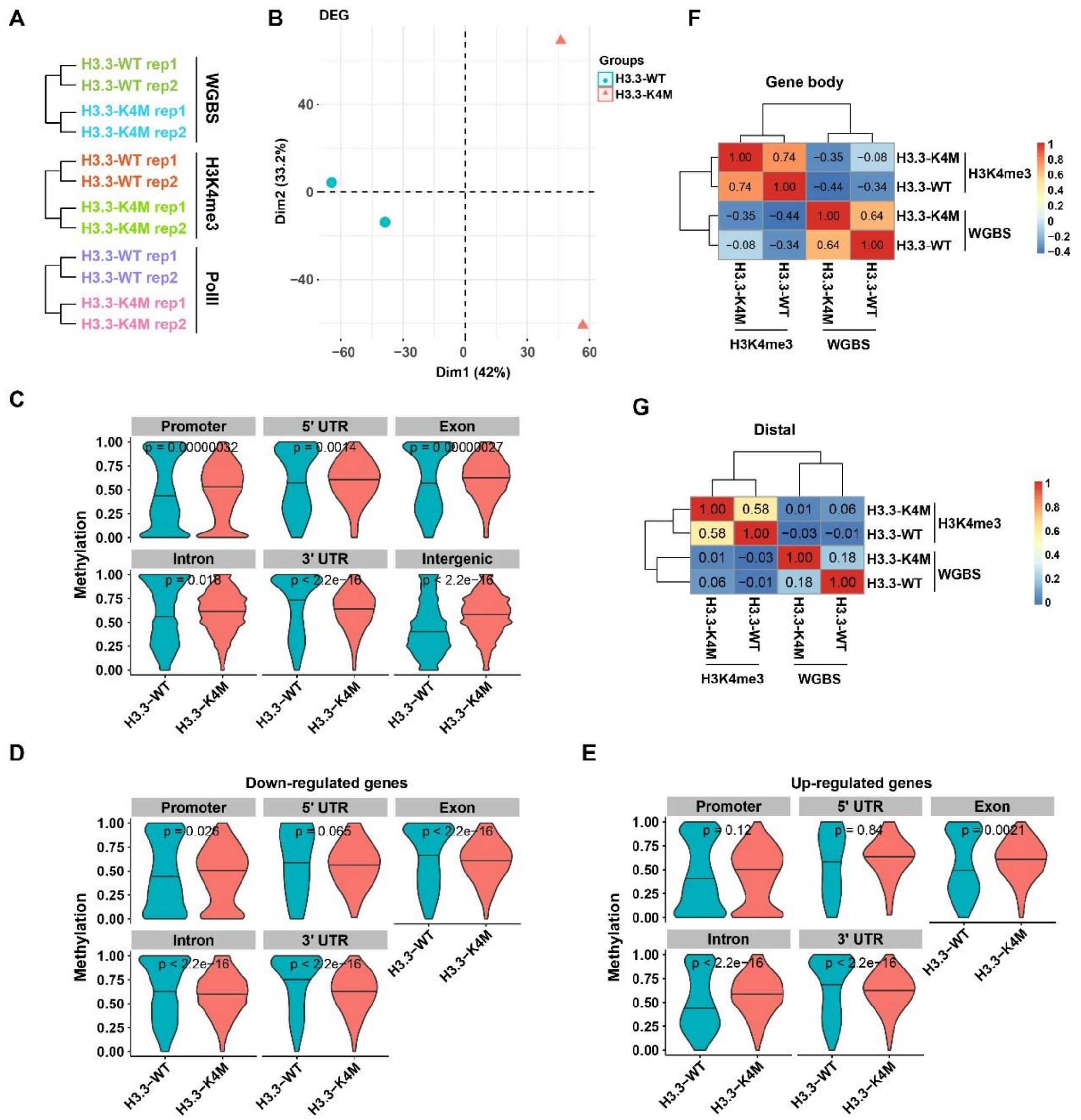

Fig. S4. DNA methylation analysis of H3.3-WT/K4M oocytes.

(A) Hierarchical clustering of DNA methylation, H3K4me3 enrichment and Pol II enrichment at gene promoter regions by Pearson correlation coefficient analysis. (B) PCA analysis of global DNA methylation of H3.3-WT and H3.3-K4M oocytes. (C) Violin plot shows alterations of DNA methylation at gene feature regions including promoter, 5’UTR, exon, intron, 3’UTR and intergenic region, with black horizontal lines indicating the median. P values by Wilcoxon Test. (D) Violin plot shows alterations of DNA methylation at gene feature regions of down-regulated genes, including promoter, 5’UTR, exon, intron, 3’UTR and intergenic region, with black horizontal lines indicating the median. *P* values by Wilcoxon Test. (E) Violin plot shows alterations of DNA methylation at gene feature regions of up-regulated genes, including promoter, 5’UTR, exon, intron, 3’UTR and intergenic region, with black horizontal lines indicating the median. *P* values by Wilcoxon Test. (F, G) Pearson correlation coefficient of H3K4me3 enrichment and DNA methylation over gene body and distal regions in H3.3-WT and H3.3-K4M oocytes.

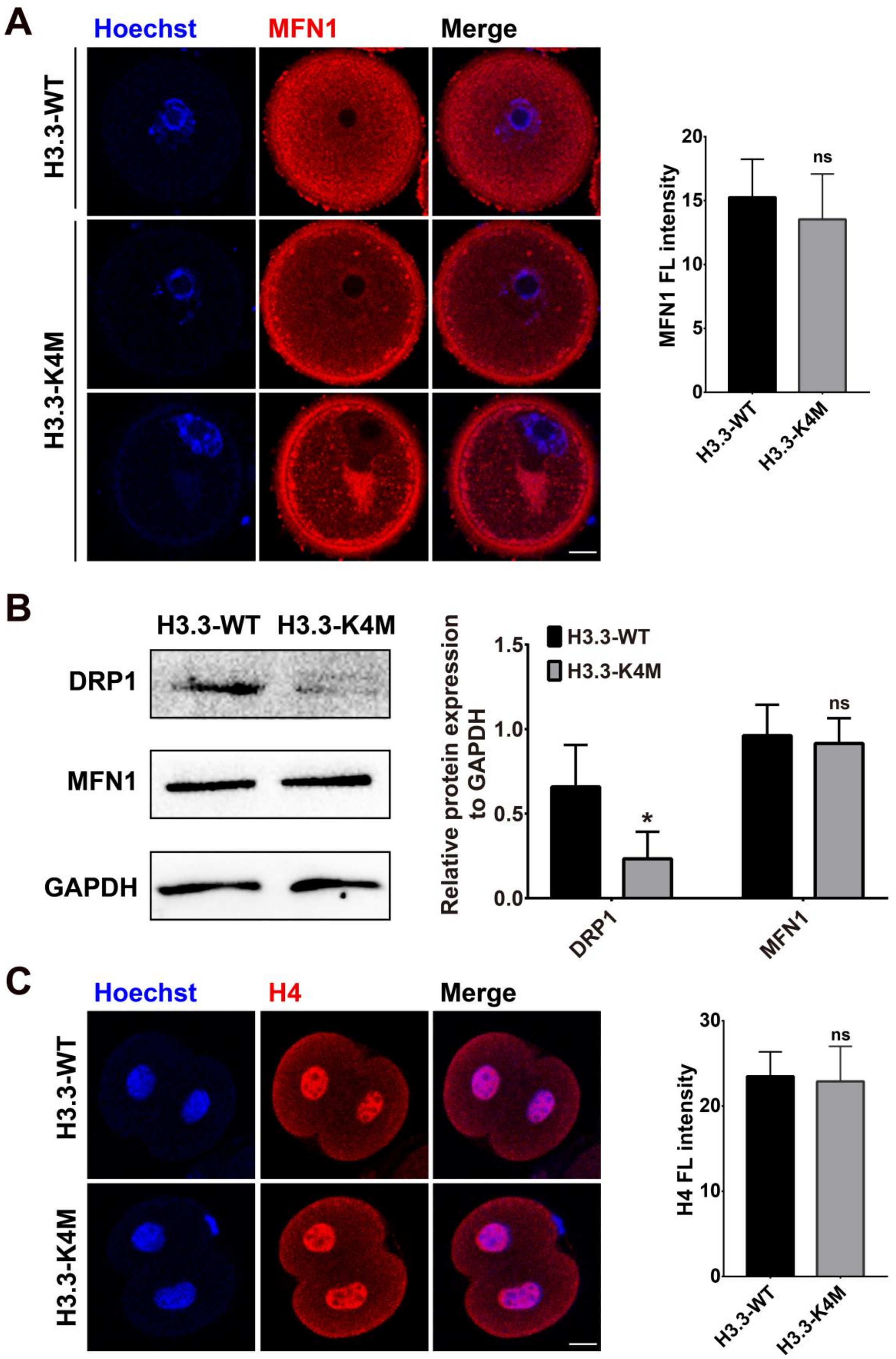

Fig. S5. Comparison of protein levels in H3.3-WT/K4M oocytes and embryos.

(A) Immunofluorescence staining and fluorescence intensities of MFN1 in H3.3-WT and H3.3-K4M GV oocytes. Scale bar=20 μm. Data are presented as Mean±SD, n=15 GV oocytes

derived from each genotype. (B) Western blot assay for intensity quantification showed abundance of DRP1 and MFN1 in H3.3-WT and H3.3-K4M GV oocytes. Data are presented as Mean±SD (n=100 for each group), $*P<0.05$. (C) Immunofluorescence staining and fluorescence intensities of histone H4 in H3.3-WT and H3.3-K4M 2-cell embryos. Scale bar=10 μm. Data are presented as Mean±SD, n=10 2-cell embryos derived from each genotype.
